# Feasibility of Little Cherry/X-Disease Detection in *Prunus avium* Using Field Asymmetric Ion Mobility Spectrometry

**DOI:** 10.3390/s25072034

**Published:** 2025-03-25

**Authors:** Gajanan S. Kothawade, Lav R. Khot, Abhilash K. Chandel, Cody Molnar, Scott J. Harper, Alice A. Wright

**Affiliations:** 1 Department of Biological Systems Engineering, Center for Precision and Automated Agricultural Systems, Washington State University, Pullman, WA 99163, USA; gajanan.kothawade@wsu.edu; 2 Department of Biological Systems Engineering, Virginia Tech Tidewater AREC, Suffolk, VA 23437, USA; abhilashchandel@vt.edu; 3 Department of Plant Pathology, Washington State University, Prosser, WA 99350, USA; cody.molnar@wsu.edu (C.M.); scott.harper@wsu.edu (S.J.H.); 4 Sugarcane Research Unit, USDA-ARS, Houma, LA 70360, USA; alice.wright@usda.gov

**Keywords:** sweet cherries, little cherry disease, post-harvest, volatile organic compounds, FAIMS

## Abstract

Little cherry disease (LCD) and X-disease have critically impacted the Pacific Northwest sweet cherry (*Prunus avium*) industry. Current detection methods rely on laborious visual scouting or molecular analyses. This study evaluates the suitability of field asymmetric ion mobility spectrometry (FAIMS) for rapid detection of LCD and X-disease infection in three sweet cherry cultivars (‘Benton’, ‘Cristalina’, and ‘Tieton’) at the post-harvest stage. Stem cuttings with leaves were collected from commercial orchards and greenhouse trees. FAIMS operated at 1.5 L/min and 50 kPa, was used for headspace analysis. Molecular analyses confirmed symptomatic and asymptomatic samples. FAIMS data were processed for ion current sum (I_sum_), maximum ion current (I_max_), and area under the curve (I_AUC_). Symptomatic samples showed higher ion currents in specific FAIMS regions (*p* < 0.05), with clear differences between symptomatic and asymptomatic samples across compensation voltage and dispersion field ranges. Cultivar-specific variation was also observed in the data. FAIMS spectra for LCD/X-disease symptomatic samples differed from those for asymptomatic samples in other Prunus species, such as peach and nectarines. These findings support FAIMS as a potential diagnostic tool for LCD/X disease. Further studies with controlled variables and key growth stages are recommended to realize early-stage detection.

## 1. Introduction

Sweet cherry (*Prunus avium*) is an economically significant stone fruit mostly grown in the Pacific Northwest (PNW) region of the United States [1,2]. Since 2010, this industry has been beset by the parallel outbreaks of little cherry disease (LCD) and X-Disease, caused by either Little Cherry virus-2 (LChV2) or ‘*Candidatus* Phytoplasma pruni’ (Ca. P. pruni), commonly referred to as the X-disease phytoplasma (XDP) [3,4]. Both pathogens adversely affect fruit maturation with smaller size and poor color. Both pathogens are phloem-limited and spread by cultural practices of propagating infectious material or by natural root grafting. LChV2 is transmitted by the apple (*Phenacoccus aceris*) and grape (*Pseudococcus maritimus*) mealybugs and ‘Ca. P. pruni’ by at least five species of leafhopper from the subfamily Deltocephalinae [5].

The most effective management option is the removal of the infected trees to limit the spread. As trees mature over several years, their removal is a substantial capital loss to the growers [6]. Unfortunately, these diseases are now prevalent throughout Washington and PNW orchards, affecting sweet and sour cherry cultivars. It is estimated that LCD/X-disease has resulted in 65$ million in losses due to trees being removed during 2015–2020 [7].

Existing detection methods include visual scouting and molecular analysis [3]. Visual scouting is often too late as the infected trees express symptoms at fruit maturation, i.e., about two weeks before the harvest stage [8,9]. Scouting is laborious and practically limited for preventive or management actions during the in-season. Molecular analysis techniques, such as Northern blot analysis, reverse transcription-polymerase chain reaction (RT-PCR), reverse transcription recombinase polymerase amplification (RT-RPA), RT-qPCR, and qPCR have also been used for LCD/X-disease detection [3,10,11,12,13]. The molecular analysis techniques can be used to detect LCD/X-disease at any growth stage as they detect pathogen-specific nucleic acids regardless of visible symptoms. They are possibly limited by the choice of sampling location as titer levels vary across the tree and may compromise diagnostic reliability [4]. These approaches are also laborious and require skilled human resources. Other potential detection methods could be the use of optical and chemical sensors. Optical sensing-based disease detections have been remarkably successful in other tree fruit crops but may have limited applicability here as LCD/X-disease infection does not lead to visible foliar symptoms [14,15,16]. Chemical sensing could potentially detect changes in the plant’s volatile organic compounds (VOCs) as a proxy for the symptoms before visual symptom expression.

Volatile sensing has been explored successfully for disease detection in fruit crops (citrus, apples and pears) and can be an option for LCD/X-disease detection. It is known that trees do emit VOCs that are intrinsic to their type, phenological stages, and health status [17,18]. Phytopathogens and insect vectors can induce changes in a plant’s VOC profile, but environmental stressors can also alter volatile production [18,19,20]. For example, distinguishable VOC signatures were observed in healthy and *C. Liberibacter* (causal agent of greening) inoculated citrus trees [21,22]. Also, some phytoplasma-infected plants are known to produce significantly higher concentrations of ethyl benzoate and an unidentified sesquiterpene than healthy apple and pear plants [23]. The VOC release patterns, type of compounds, and concentrations can vary for trees growing in biotic and abiotic stressed conditions [17,24,25,26]. The plant-pathogen interaction can significantly affect VOC release [23]. Thus, high throughput sensing of these VOCs could be instrumental for prompt disease detection and management [24,26,27]. This study evaluates this hypothesis for LCD/X-disease-associated symptoms detection in sweet cherry stem samples.

Typically, volatile biomarkers can be quantified using the headspace analysis performed by gas chromatography-mass spectrometry (GC-MS) and variants of electronic nose-type systems that use an array of gas sensors [28]. Gas sensors either use metal oxide, conducting polymer, carbon nanotubes, or affine-binding proteins deposited on substrates to sense VOCs [29,30]. Cellini et al. [31] used GC-MS to detect fire blight and blossom blight in apple plants. An e-nose equipped with ten metal oxide semiconductor chemical sensors was used to detect fungal contamination (*Botrytis cinerea*, *Monilinia fructicola*, and *Rhizopus stolonifer*) in peaches [32]. Although GC-MS can be a reliable approach, it is non-portable and requires expert personnel to perform the analysis [18,26,33]. Lower sensitivity and specificity relative to GC-MS and molecular methods [31] are added limitations of e-nose-type sensors. Field asymmetric ion mobility spectrometry (FAIMS) that works on the principle of separating gas ions based on differences in their mobility under high-electric fields [34] could be a potential high throughput sensing solution in such scenarios. Ideally, FAIMS can provide a distinct VOC fingerprint for a given sample under controlled conditions [35]. The spectral response, however, could be governed by the testing environment and sample inference by the surrounding agricultural ecosystem. Detailed working and example applications of FAIMS in agricultural systems are summarized by Kafle et al. [18], Sinha et al. [36,37,38], and Kothawade et al. [33,39]. Portable FAIMS systems have recently emerged as rapid analytical tools to detect volatile profiles associated with post-harvest diseases like potato tuber rot and onion sour skin [33,36,37,38,39]. Specific to fruit crops, FAIMS and GC-differential mobility spectrometry (DMS) have been explored for the citrus Huanglongbing disease detection [21,22]. Citrus tree samples with varying symptoms were collected and analyzed using GC-DMS. Trees were categorized into healthy and infected groups based on the top five most discriminating volatile compounds with >90% accuracy.

The above studies successfully demonstrated the potential of FAIMS for disease detection. However, no prior study has investigated the feasibility of the FAIMS-based volatile headspace sampling technique for detecting LCD/X-disease infection in cherries. This study explores this gap with specific objectives: (1) to evaluate the feasibility of LCD/X-disease detection using FAIMS and compare the results to qPCR-based molecular analysis in three sweet cherry cultivars: ‘Benton’, ‘Cristalina’ and ‘Tieton’, (2) to identify the key features associated with the LCD/X-disease symptoms and, (3) to investigate the diagnostic potential of FAIMS-based detection in other host *Prunus* species, such as peach (*Prunus persica*) and nectarine (*Prunus persica* var. *nucipersica*). Efforts focus on sampling at the post-harvest growth stage as the titer level is maximum and coincides with expressed disease symptoms. We hypothesize that if volatile biomarkers are distinguishable at this stage, future studies can be conducted toward pre-symptomatic detection.

## 2. Materials and Methods

### 2.1. Field Sampling

**Figure 1:**
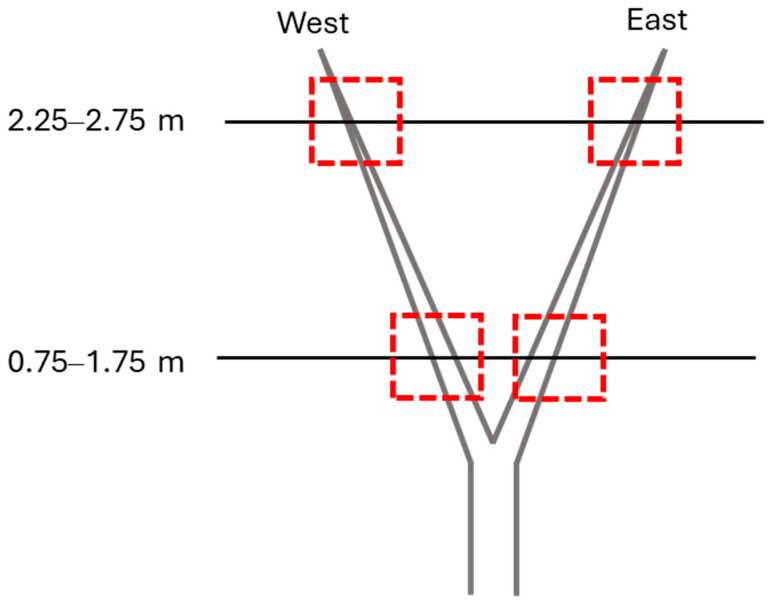
Sampling locations above ground level (AGL) on the orchard-grown cherry trees (cv., ‘Benton’; training system: Tatura). Red boxes represent the sampling locations on the tree.

In this study, stem samples were collected at the post-harvest growth stage of the 2021 season from the sweet cherry trees grown in commercial orchard blocks located in central Washington as well as from greenhouse-grown trees (source: WSU Clean Plant Network). The total number of sampled trees was based on the availability of symptomatic (S) and asymptomatic (AS) trees in the orchard block and greenhouse. Samples were collected from three cultivars, namely, ‘Benton’ (S: 12 [3 trees × 4 replicates]; AS: 14 [3 trees × 4 replicates; 2 greenhouse trees × 2 replicates]), ‘Cristalina’ (S: 3; AS: 3), and ‘Tieton’ (S: 3; AS: 2). The samples were collected around 10:00 am on a non-spray day to avoid any noise coming from chemical applications. The ‘Benton’ cultivar trees were trained on the Tatura trellis system [40]. The ‘Cristalina’ and ‘Tieton’ cultivar trees had a free-standing training system. For the ‘Benton’ cultivar, samples were collected from the top (2.25–2.75 m above ground level, AGL) and bottom (0.5–0.75 m AGL) zone of the east and west sides of the tree (Figure 1). Preliminary statistical analysis (*t*-test) for Benton cultivar showed separation between ion currents for AS and S samples collected from lower canopy zones. Therefore, samples for the ‘Cristalina’ and ‘Tieton’ cultivars were collected from the lower canopy zone of the trees (0.5–0.75 m AGL). A total of 37 samples (one/tree), each containing four stems, were collected and stored in an ice chest before further processing.

An equal number of samples were also collected from the same trees and same branches for conducting qPCR-based molecular analysis [3] as an independent validation of the FAIMS-based analysis. The qPCR method was selected for its high sensitivity, specificity, and ability to provide quantitative data and prior proven results to detect LCD/X disease. Similarly, samples were collected from commercial peach (cultivar: ‘Diamond Princess’; S: 5; AS: 5) and nectarine (cultivar: ‘Due South’; S: 5; AS: 2) blocks for validating the diagnostic potential of FAIMS-based detection in other *Prunus* species.

### 2.2. Volatile Headspace Analysis

**Figure 2:**
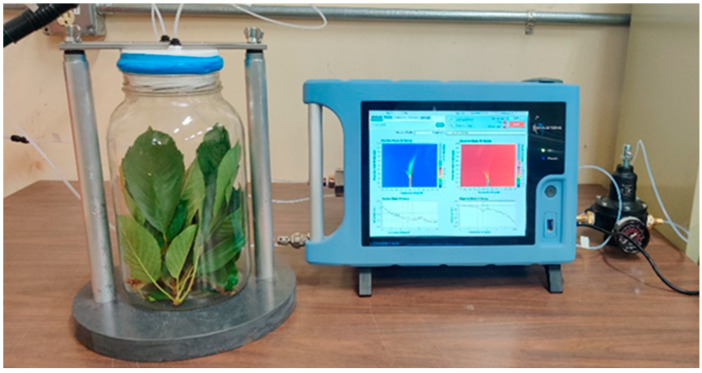
Portable FAIMS-based volatile headspace sampling setup for LCD/X-disease detection.

Field-collected samples were transferred to sterilized 1-gallon glass jars (Specialty Bottle, Seattle, WA, USA). The jars were then covered with an oxygen-permeable food-grade cling film and stored at 25 °C for 2 h for volatile headspace accumulation. Humidity levels were not actively monitored but were consistent throughout the sampling process. Also, a scrubber in the FAIMS inlet column absorbs any moisture in volatile headspace to minimize noise in the data. Volatile headspace sampling was then conducted for each jar using a portable FAIMS system (Lonestar, Owlstone Medical Ltd., Cambridge, UK). The volatile headspace is the gaseous phase above a sample, containing vaporized organic compounds in equilibrium with the sample matrix. Nitrogen was used as the carrier gas due to its inert nature and to avoid contamination and interference from ambient air. During sampling, each jar was covered with a Teflon lid having two stoppers with holes, one for the carrier gas inlet and another for the headspace and carrier gas mixture outlet to the FAIMS ionization chamber (Figure 2). A total of six FAIMS scans were recorded for each sample jar at the optimized operational parameters (flow rate: 1.5 L min^−1^ and pressure: 60 kPa). The system was then purged for about 30 min using the carrier gas before sampling the next sample jar (cleaning step). The purging process was performed to ensure the complete removal of residues between measurements to avoid cross-contamination. The sampling sequence was randomized to avoid statistical bias [39]. A blank jar was also scanned as a reference for each experiment of volatile sampling.

In total, 228 FAIMS scans (37 samples [37 tree samples] × 6 scans/samples) were obtained. The FAIMS spectra provide a three-dimensional dataset consisting of compensation voltage (CV), dispersion field (DF), and ion current (I_c_) data. Each scan included both positive and negative polarity spectra, consisting of I_c_ spectra proportional to the mass of distinct released VOCs under a range of CV: −6 to 6 V and DF intensities of 0% to 100%. Each scan was stored in a three-dimensional dataset consisting of 512 CVs, 51 DFs, and resultant I_c_ (arbitrary units, AU). The FAIMS system outputs a normalized signal. The range and number of CVs and DFs were selected based on the manufacturer’s guidelines. Theoretically, the selection of 512 CVs (step size: 24 mV) settings provides a fine granularity in tuning the electric field to compensate for the ion mobility under the applied DF. The 51 DFs from 0% to 100% of the maximum dispersion field cover the full range of this parameter.

### 2.3. FAIMS Data Analysis

FAIMS dispersion field matrix files in default ‘.dfm’ file format were converted to ‘.txt’ file format using the system’s software, ‘Review DF Matrix Offline’ (Owlstone Medical Ltd., Cambridge, UK). A custom-developed Python (version 3.10.8, Python Software Foundation, Wilmington, DE, USA) script was then used to read the ‘.txt’ files and organize the data of CV, DF, and positive I_c_ associated with each scan in a ‘.csv’ file format [39]. Based on findings from prior studies, only positive spectra were considered for analysis, as negative spectra did not yield any useful information [37,39]. Out of six scans, only the third and fourth scans were considered stable scans and used for further analysis [33]. The final dataset had 26,112 rows (CV-DF combinations) and 78 columns (CV, DF, and 78 scans [37 samples × two scans/sample]) for the entire sampling.

**Figure 3:**
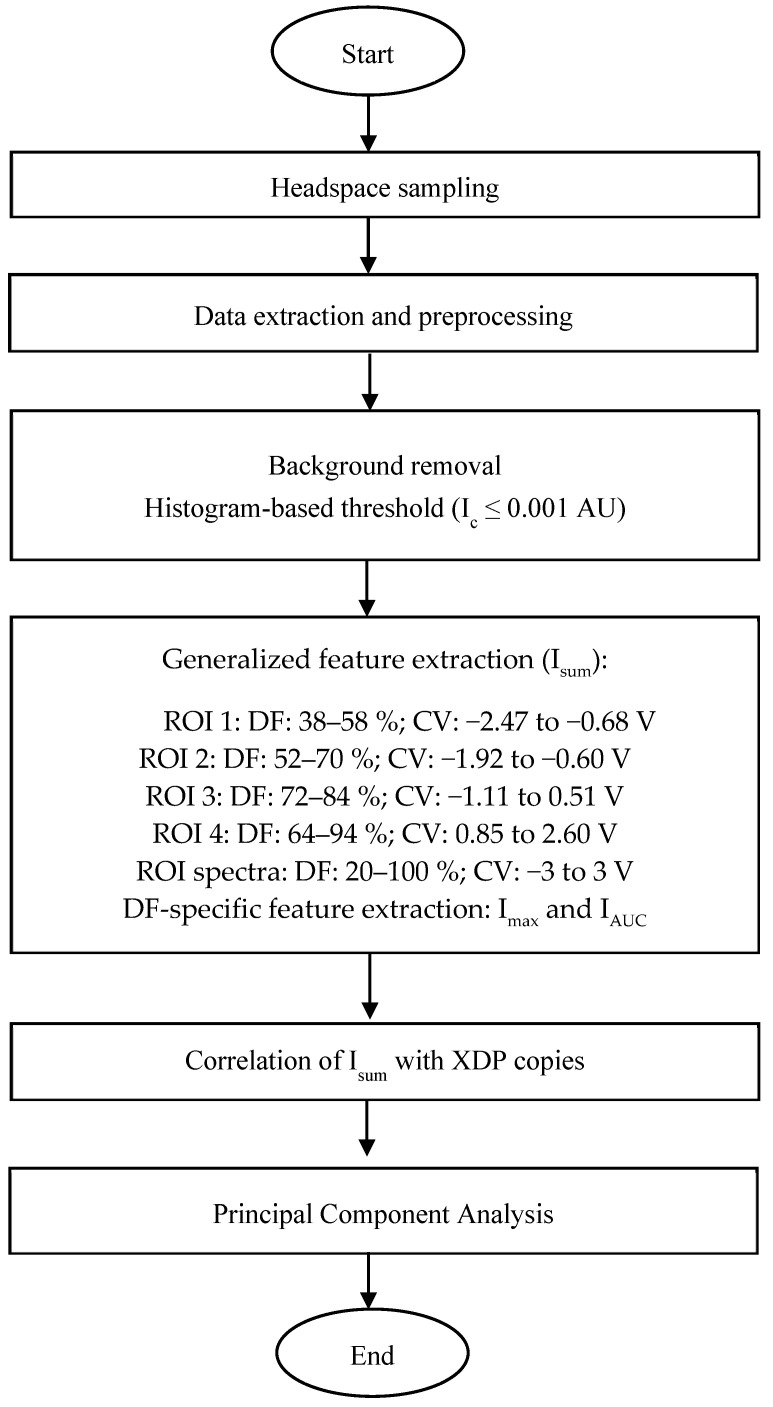
FAIMS data analysis pipeline for LCD/X-disease detection. I_c_: Ion current; AU: Arbitrary unit; I_sum_: Ion current sum; ROI: Region of interest; DF: Dispersion field; CV: Compensation voltage; V: Volts; I_max_: Maximum ion current; I_AUC_: Area under the curve; XDP: X-disease phytoplasma.

Preliminary dataset visualization indicated the presence of four I_c_ peaks in the samples. The third I_c_ peak was distinct and was present in infected samples. In general, the signals from distinct substances in FAIMS data overlap, resulting in a noisy signal. Noise removal and feature engineering were thus performed to identify the important features. A threshold value (I_c_ > 0.02) was determined using histogram-based thresholding and used for filtering the noise. Based on the I_c_ clusters, a total of four generalized regions of interest (ROIs) were defined and used to extract the sum of I_c_ (I_sum_) at all CV-DF combinations within the respective ROIs (Figure 3). I_sum_all_ as the sum of the entire spectra was also computed as a feature. Pearson and Spearman correlation analysis was performed on the I_sum_ features, and the number of XDP copies was calculated by molecular analysis. Additional features, maximum ion current (I_max_) and area under the curve (I_AUC_) were extracted at each DF within a given CV range for the DF-specific analysis. I_max_ is the highest ion current measured for any given DF within a specified range of CV values. A scanline at a given DF value and CV range is a 2D representation of the spectra. The I_AUC_ was then calculated by integrating the I_c_ over a specific range of CV at a given DF using the trapezoidal method. For each DF value, these features were calculated and compressed along with the sample names as rows, DF values in columns, and respective I_AUC_ values. Principal component analysis (PCA) was then performed on the extracted features for pattern recognition. The number of phytoplasma copies in the sample was calculated using a calibration curve and quantification cycle (Cq) values [4]. Comparative analysis was performed by correlation analysis between important features and the number of phytoplasma copies in a sample. All the analyses and visualizations were conducted in Python software packages using libraries, ‘NumPy’, ‘pandas’, ‘seaborn’, ‘scikit-learn’ and ‘matplotlib’.

## 3. Results and Discussion

The number of XDP copies in the samples varied from 118,928 to 1,833,537 for the ‘Benton’, 144,046 to 278,907 for ‘Cristalina’, and 377 to 1412 for ‘Tieton’ cultivar, which is within the range that symptoms are commonly present. The rapidity in which different *Prunus* species develop symptoms, their type and severity of symptoms presented depend on the infecting X-disease phytoplasma genotype, the species or cultivar infected, phytoplasma titer, and how far the infection has progressed [3,41]. It has been reported that XDP is unevenly distributed in the host plants [4]. The titer level of LCD/X disease also varies between early and established (or visible) infection stages and across the seasons [41].

### 3.1. Salient FAIMS Spectra

**Figure 4:**
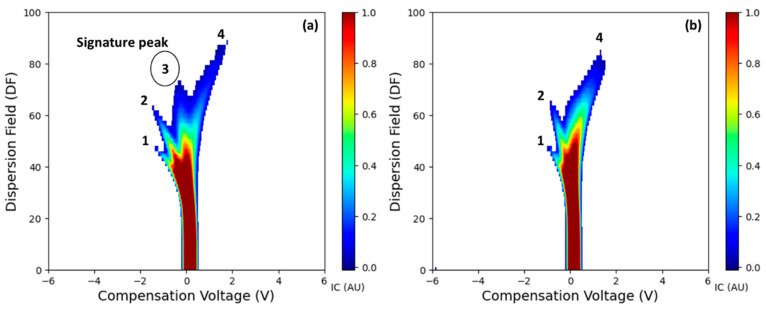
FAIMS spectra with volatile headspace signature specific to (**a**) LCD/X-disease symptomatic and (**b**) asymptomatic samples for the ‘Benton’ cultivar. Numbers 1–4 correspond to the ion current peaks in the spectra. The potential signature peak observed in infected samples is highlighted with a circle.

Typical spectra (positive polarity) for representative LCD/X-disease S and AS samples are shown in Figure 4. Three I_c_ peaks (clusters) were observed in AS samples while an additional peak curving to the left and then top (labeled as signature peak; CV: −0.11 to 0.51 V and DF: 64–84%) was present in S samples (Figure 4). This peak appeared to be uniform for the ‘Benton’ (Figure 4) and with variations for ‘Cristalina’ and ‘Tieton’ cultivars (Figure S1). Such distinct peaks in S samples could be associated with volatile release and may be attributed to the plant defense pathways in response to phytoplasma colonization [42]. This peak was also present with very low intensity in some of the AS samples, which were tested at an AS titer level (early infection, Cq > 30). Previous FAIMS feasibility studies in post-harvest disease detection also reported a distinct peak associated with the disease infection [33,36,37,38,39]. FAIMS applications in medical diagnostics also observed the presence of one or more distinct peaks in the spectra from infected samples [35,43].

The first two I_c_ peaks, numbered 1 and 2 in Figure 4a,b, are potentially the reactant ion peaks formed after the ionization of nitrogen carrier gas [38,39]. In the FAIMS analysis of a sample, the carrier gas is ionized by the radioactive source (Ni-63), which initially ionizes the nitrogen molecules. Ionization results in the initial I_c_ peaks in a low electrical field [44] and could be related to the RIPs at lower regions in the spectra. Intense spectra (higher I_c_ values) for RIPs were observed in very few AS and S samples. Interestingly, these samples were from the top canopy zones. The I_c_ intensity and the shape of the fourth peak, starting from the center and curving towards the right corner (CV: 0.85 to 2.60 V; DF: 64–94%), were also observed to be different for the S and AS samples (Figure 4 and Figure S1). I_c_ values and distribution of fourth peak had a separate presence in the S samples and a compact pattern in the AS samples of ‘Benton’ cultivar. Similar patterns with varying I_c_ intensity were observed in the ‘Cristalina’, whereas a peak with a small shape was present in the ‘Tieton’ cultivar samples (Figure S1)

### 3.2. Feature Selection

**Figure 5:**
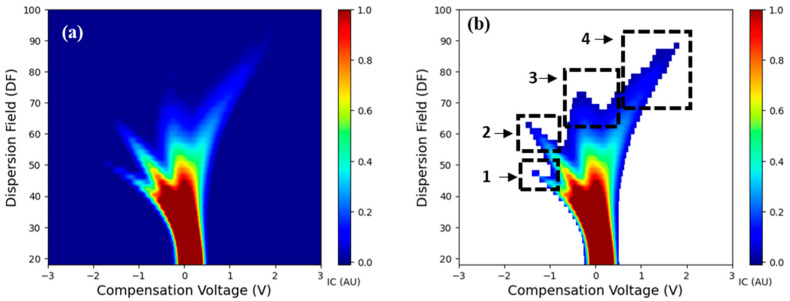

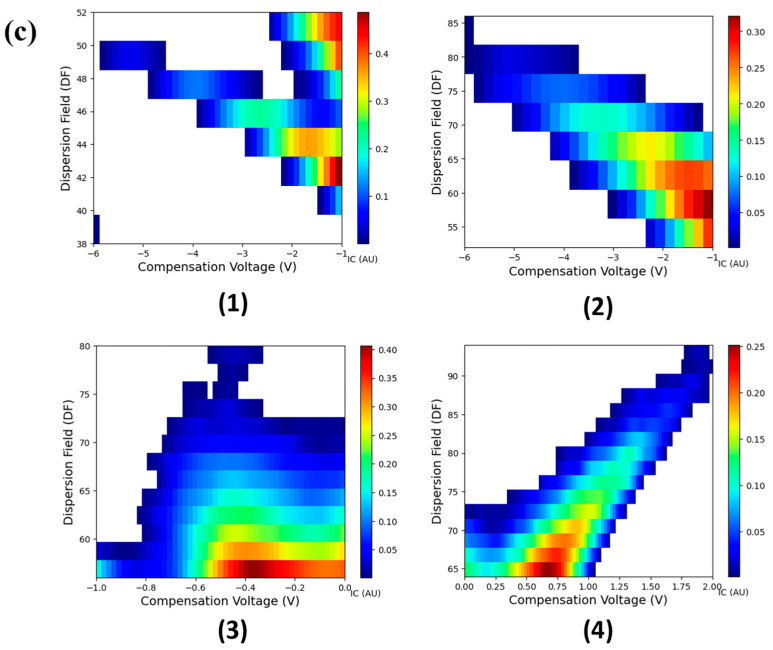
ROI selection and feature extraction process for FAIMS spectra: (**a**) raw spectra, (**b**) filtered spectra, and (**c**) extracted ROIs (1–4 attributes to the peak ID).

Plants release different blends of VOCs, and the resultant FAIMS spectra could be a combination of compounds that may produce different spectra with varying shapes and intensities. Distinct I_c_ clusters were observed, but feature engineering was employed to extract the features to quantify this distinction. Statistical analysis specific to CV-DF features may have the possibility of false detection (Tables S1–S5). Therefore, features were selected based on the I_c_ clusters and spread within respective CV-DF ranges. The shape and intensity of these peaks were observed to be different in all the samples, possibly because each biological sample and associated secondary metabolism is different. This variability was addressed by considering generalized ROIs for the peaks. The derived feature, I_sum,_ from the extracted ROIs was calculated and considered as a feature that represents the peak as a bulk (Figure 5). Additionally, the I_sum_ data for the entire filtered spectra was calculated and added as an additional feature to the data set.

Overall, intra- and inter-specific variations were evident in the I_c_ spectra for all the samples (*p* < 0.05; Table S6). The shape and intensity of the signature peak were prominent for the ‘Benton’ cultivar compared to the other two cultivars (based on I_max_ values). Inter-cultivar differences were also evident in the I_c_ peaks for samples from the same cultivar and could be attributed to differences in titer levels, foliage thickness, and tree age [22]. Phytoplasma infection often alters chlorophyll, sugar, and secondary metabolite contents, which, in turn, alter source-sink relationships at the plant’s biochemical level and affect spectral response [45]. Correlation analyses using both Pearson (*r*: 0.06–0.27; *p*: 0.17–0.78) and Spearman (*ρ*: 0.04–0.41; *p*: 0.19–0.83) methods outlined that I_sum_ features do not exhibit linear or monotonic association with the number of XDP copies in the samples. These results suggest that there is not enough evidence to reject the null hypothesis of no significant correlation between I_sum_ features and XDP copies. The distance between the sampling location and the inoculation site within a tree could also potentially impact the behavior of phytoplasma in the plant tissue.

**Figure 6:**
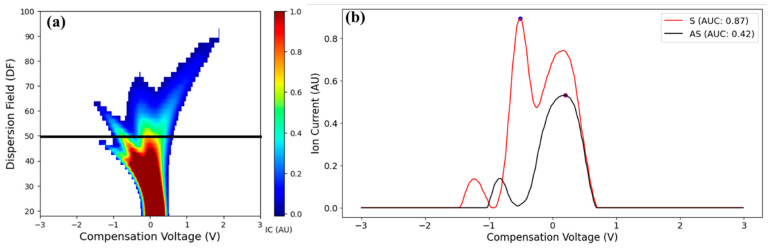
DF-specific feature extraction: (**a**) FAIMS maximum Ion current and (**b**) area under the curve estimation.

The typical I_max_ and I_AUC_ feature extraction process is illustrated in Figure 6. DF line plots revealed the patterns of S and AS samples. When the data along the CV was visualized for all the samples, a pattern specific to the CV range was observed between both sample groups (Figure S2, pattern highlighted with sample group labels). This suggests that the I_c_ intensities pertinent to the LCD/X-disease positive samples have distinct peaks in the CV range of −2.0 to 0.0 V. Whereas distinct I_c_ peaks were observed for LCD/X-disease AS samples in the CV range of 0.0 to 2.0 V. This observation supports the presence of the third peak in the positive samples. The differences between the red (S samples) and green (AS samples) lines suggest that the infection changes the I_c_ profile, which could be due to the presence of different VOCs or the metabolic products associated with the LCD/X-disease infection. Additionally, the spread and intensity of I_c_ peaks at different CVs suggest that there are different ionizable species present in S versus AS samples. The ion current cluster variations between the signature peaks for positive samples could also be due to the extent of the pathogenic distribution along the tree.

### 3.3. Pattern Recognition

**Figure 7:**
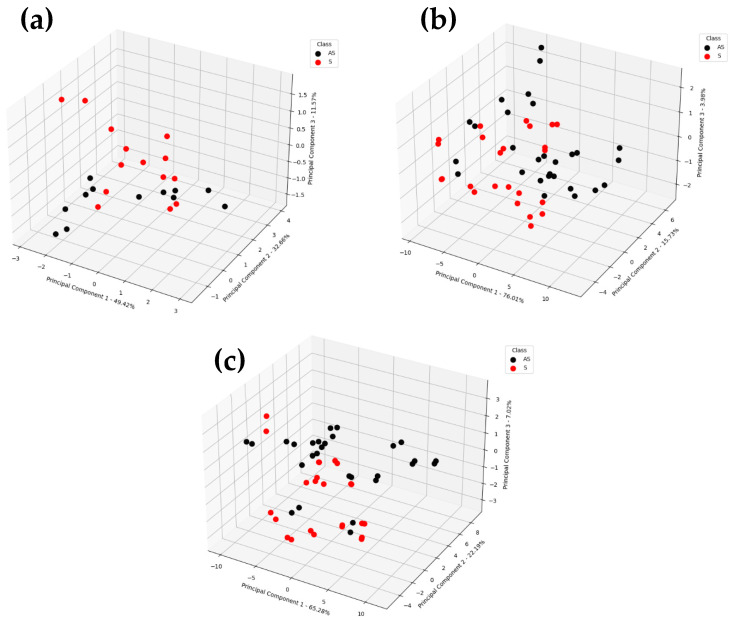
Principal component analysis biplot for the features: (**a**) ion current sum; (**b**) area under the curve; (**c**) max. ion current within the given CV-DF range. The numbers following the en dash represent the percentage of variance explained by the corresponding principal components.

The PCA revealed that most of the variation (>90%) was captured by the first three PCs. The derived I_c_ patterns were not clearly distinct from each other but did show separation. Three different clusters were observed in the PCA biplots (Figure 7). Two different clusters were observed within the AS datapoints and could be attributed to the biological variation between the samples as well as the spatial distribution of VOCs for samples from different locations within a tree, i.e., upper, and lower canopy zones and east/west sides of the canopy (Figure 7). The AS greenhouse samples appeared separated from the main cluster, which could be attributed to the smaller Ic spectra (Figure 7). Compact data points (two points around one location) within the cluster represent the multiple scans (scans 3 and 4) of the same sample, indicating a similar VOCs release pattern [39]. These clusters could possibly be grouped based on the level of infection as undetected, early infection (Cq > 30), and established infection (Cq < 30). Movement of the phytoplasma is generally basipetal towards the roots, although it may be transported to a local sink near the inoculation site depending on the strength of the sink pressure [5,41,46]. The intensity of VOCs may also vary between samples due to different foliage thicknesses, leaf size [47] and the time-of-day samples were collected [22]. Another artifact could be related to the oxidative stress in the samples [48].

### 3.4. Spatial Distribution of VOCs

The volatile release pattern was observed to be different between the biological replicates from the top and bottom canopy zones as well as the east and west sides of the trees. The LCD/X-disease-associated I_c_ cluster was observed consistently in the infected samples, more specifically in the samples collected from the lower canopy zone. Wright et al. [4] studied the spatial distribution of ‘Ca. P. pruni’ titer at two main scaffolds facing east and west at different heights, with branches of the scaffold pointing north and south. The trees were grouped for analysis based on the pattern of X-disease phytoplasma distribution. The study reported that there is a definite spatial pattern to ‘Ca. P. pruni’ distribution in cherry trees changes over time; the trees sampled in this FAIMS study were selected from this same set of trees.

Tree-specific variation was observed in the replicates of the same tree. The RIP spectra (ROI 1 and 2, Figure 5) were observed to be more intense for the upper canopy zone than the lower canopy zone samples. The appearance of the third I_c_ peak was not consistent in the samples collected from the top canopy zones. This could be due to the phytoplasma movement in a basipetal direction from the roots to all the parts of the tree through the phloem. As lower branches are closest to the roots, they could have experienced more impact than the top branches. It was not possible to validate if the observed trend continued, as the grower removed the trees following the 2021 harvest.

### 3.5. Validation with Other Host Species of LCD/X-Disease

**Figure 8:**
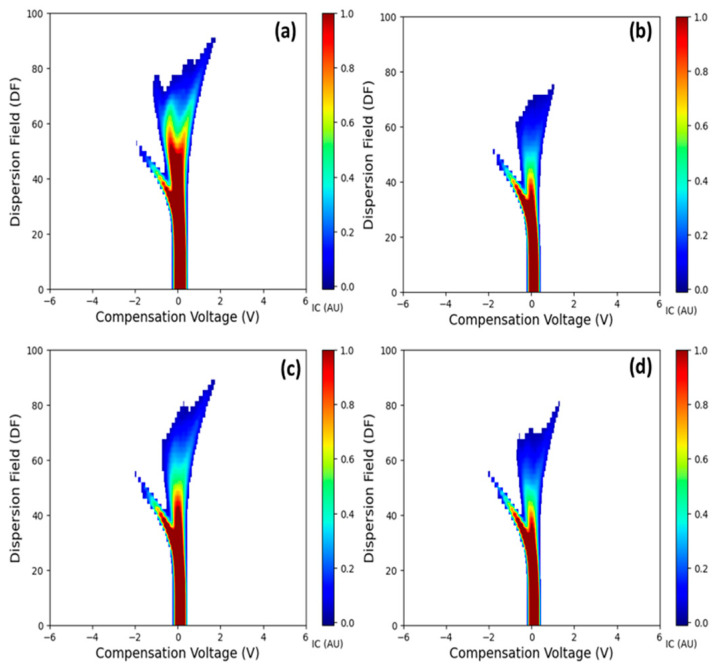
Volatile headspace signatures are specific to LCD/X-disease positive (XDP) (**a**) peaches, (**c**) nectarines and asymptomatic (AS) samples for (**b**) peaches, (**d**) nectarines.

The FAIMS spectra in the LCD/X-disease infected samples were observed to be distinct from the AS samples for the other host species, such as peach and nectarines (Figure 8). While LCD/X-disease infection in peaches and nectarines exhibits visual symptoms on both leaves and fruits, and trees may exhibit decline with time and eventually die [5]. The FAIMS spectra obtained from these hosts show distinct characteristics when compared to the spectra of sweet cherry samples. The maximum I_c_ values in both peaches and nectarines were lower than those observed in sweet cherry samples. Specific to peach samples, a distinct peak was observed in S samples and could be a signature peak for LCD/X disease. This confirms the diagnostic potential of FAIMS-based detection in other host species of these diseases. While similar observations could be inferred from the nectarine samples, the limited number of samples prevented further conclusions from being drawn.

## 4. Conclusions

1. The FAIMS system detected differences in the VOC profiles between LCD/X-disease symptomatic and asymptomatic sweet cherry samples for ‘Benton’, ‘Cristalina’ and ‘Tieton’ cultivars. A distinct third ion current peak was identified as the potential signature feature potentially associated with the disease symptoms. Overall, symptomatic samples exhibited higher ion currents compared to the asymptomatic ones for features like I_max_ and I_AUC_ across different DF intensities.
2. PCA revealed clustering in the FAIMS data, suggesting potential differentiation between infection levels and sample types.
3. VOC profiles varied across cultivars, possibly due to intrinsic biological differences and varying pathogen titer levels. Despite most cherry cultivars being heavily inbred, the cultivars examined here are not closely related, suggesting that these results may be broadly applicable to all cherry cultivars [49].
4. The FAIMS spectra for LCD/X-disease infected samples were distinct from AS samples of other host *Prunus* species, such as peach and nectarines. This further confirms the diagnostic potential of the FAIMS system.

This study establishes a pathway for future research toward pre-symptomatic detection of LCD/X-disease using volatile sensing techniques. Such an approach can be instrumental for timely grower decision-making related to removing the infected trees and restricting further disease spread. Studies are warranted to evaluate FAIMS for LCD/X-disease detection in other commercially important cherry cultivars. The findings and optimized experimental parameters from this study could be further enhanced by analyzing samples at different key growth stages for pre-symptomatic stage detection at high throughput.

## Data Availability

The data presented in this study are available on request from the corresponding author.

## Supplementary Materials

The following supporting information can be downloaded at: https://www.mdpi.com/article/10.3390/s25072034/s1, Figure S1. Volatile headspace signatures specific to LCD/X-disease positive cherry samples: (a) ‘Cristalina’, (c) ‘Tieton’ and asymptomatic (AS) samples for (b) ‘Cristalina’ and (d) ‘Tieton’ cultivars. Figure S2. Volatile headspace signatures specific to LCD/X-disease positive (red) and asymptomatic (green) samples along CV for ‘Benton’ cultivar. Table S1. Ion current (Mean ± Std. Error, AU) pertaining to significant CV-DF combinations (first five) for asymptomatic and LCD/X-disease symptomatic samples of all tested cultivars. Table S2. Ion current (Mean ± Std. Error, AU) pertaining to significant CV-DF combinations (first ten) for LCD/X-disease symptomatic and asymptomatic samples for cultivar: ‘Benton’. Table S3. Ion current (Mean ± Std. Error, AU) pertaining to significant CV-DF combinations (first ten) for LCD/X-disease symptomatic and asymptomatic samples for cultivar: ‘Cristalina’. Table S4. Ion current (Mean ± Std. Error, AU) pertaining to significant CV-DF combinations (first ten) for LCD/X-disease symptomatic and asymptomatic samples for cultivar: ‘Tieton’. Table S5. Significant compensation voltage and dispersion field combinations for LCD/X-disease detection. Table S6. ANOVA for intra- and inter-specific variations in the ion current spectra for all tested cultivars. S: symptomatic; AS: asymptomatic.

## Abbreviations

LCD, Little cherry disease; FAIMS, Field asymmetric ion mobility spectrometry; CV, compensation voltage; DF, dispersion field; VOC, volatile organic compounds; LChV2, Little cherry virus 2
