# Supplementary material for: Feasibility of Little Cherry/X-Disease Detection in Prunus avium Using Field Asymmetric Ion Mobility Spectrometry
Source: Sensors (Basel). 2025 Mar 25;25(7):2034. doi: 10.3390/s25072034 (PMC11990987; doi:10.3390/s25072034)
Supplement: Supplementary file 1 [file sensors-25-02034-s001.zip › sensors-3469684-supplementary.pdf]

The Feasibility of Little Cherry/X-Disease detection in *Prunus avium* using Field Asymmetric Ion Mobility Spectrometry

Gajanan S. Kothawade[1], Lav R. Khot[1,*], Abhilash K. Chandel[2], Cody Molnar[3], Scott J. Harper[3], Alice A. Wright[4]

[1]Department of Biological Systems Engineering, Center for Precision and Automated Agricultural Systems, Washington State University, Pullman, WA 99163, USA
[2]Department of Biological Systems Engineering, Virginia Tech Tidewater AREC, Suffolk, VA 23437, USA
[3]Department of Plant Pathology, Washington State University, Prosser, WA 99350, USA
[4]Sugarcane Research Unit, USDA-ARS, Houma, LA 70360, USA

*Corresponding Author: lav.khot@wsu.edu

Supplementary

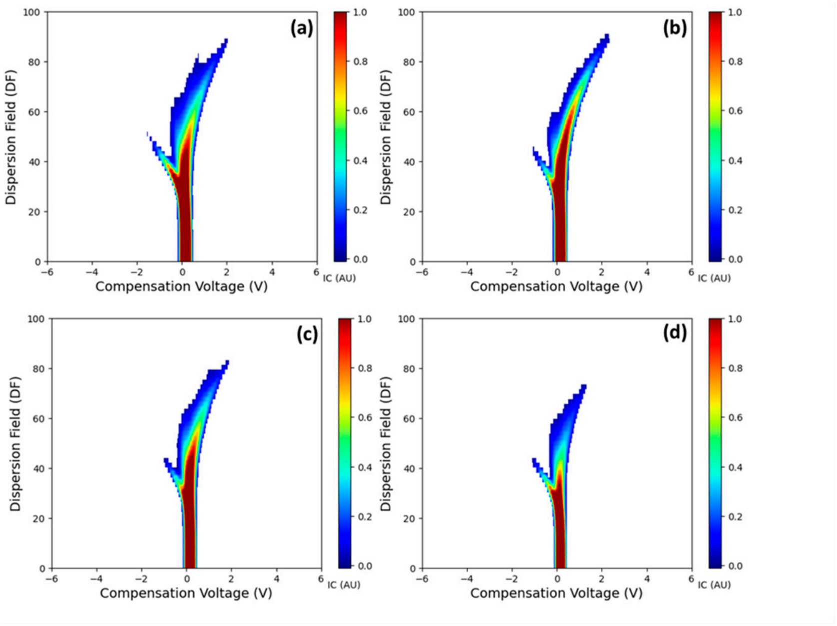

Figure S1. Volatile headspace signatures specific to LCD/X-disease positive cherry samples: (a) 'Cristalina', (c) 'Tieton' and asymptomatic (AS) samples for (b) 'Cristalina' and (d) 'Tieton' cultivars.

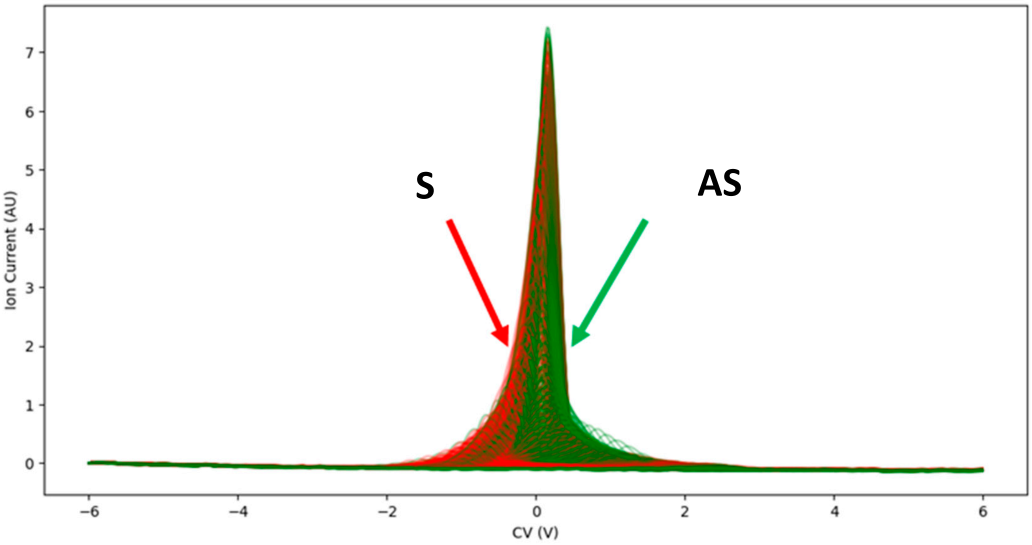

Figure S2. Volatile headspace signatures specific to LCD/X-disease positive (red) and asymptomatic (green) samples along CV for 'Benton' cultivar.

Table S1. Ion current (Mean ± Std. Error, AU) pertaining to significant CV-DF combinations (first five) for asymptomatic and LCD/X-disease symptomatic samples of all tested cultivars.

Cultivar	CV	DF	Asymptomatic	Symptomatic	p-value
Benton	0.03	78.00	-0.05 ± 0.00	-0.04 ± 0.00	< 0.00
	0.69	96.00	-0.08 ± 0.00	-0.07 ± 0.00	< 0.00
	0.69	74.00	-0.04 ± 0.00	-0.03 ± 0.00	< 0.00
	0.52	80.00	-0.05 ± 0.00	-0.04 ± 0.00	< 0.00
	0.69	90.00	-0.07 ± 0.00	-0.06 ± 0.00	< 0.00
Cristalina	0.01	72.00	-0.06 ± 0.00	-0.07 ± 0.00	0.02
	-0.67	88.00	-0.06 ± 0.00	-0.07 ± 0.00	0.02
	-0.60	82.00	-0.06 ± 0.00	-0.07 ± 0.00	0.04
	1.06	98.00	-0.07 ± 0.00	-0.08 ± 0.00	0.04
	-0.30	90.00	-0.07 ± 0.00	-0.07 ± 0.00	0.04
Tieton	-0.65	86.00	-0.08 ± 0.00	-0.07 ± 0.00	< 0.00
	-0.67	94.00	-0.08 ± 0.00	-0.06 ± 0.00	< 0.00
	-0.65	96.00	-0.08 ± 0.00	-0.07 ± 0.00	< 0.00
	1.18	70.00	-0.07 ± 0.02	0.25 ± 0.01	< 0.00
	1.18	74.00	-0.03 ± 0.00	0.17 ± 0.03	< 0.00

All numerical observations have been reported up to two decimal digits.

Table S2. Ion current (Mean ± Std. Error, AU) pertaining to significant CV-DF combinations (first ten) for LCD/X-disease symptomatic and asymptomatic samples for cultivar: 'Benton'.

**CV**	**DF**	**Asymptomatic**	**Symptomatic**	***p* -value**
-0.58	88.00	-0.09 ± 0.00	-0.08 ± 0.00	< 0.00
-0.60	88.00	-0.09 ± 0.00	-0.08 ± 0.00	< 0.00

-0.56	88.00	-0.09 ± 0.00	-0.08 ± 0.00	< 0.00
-0.09	94.00	-0.09 ± 0.00	-0.08 ± 0.00	< 0.00
-0.11	94.00	-0.09 ± 0.00	-0.08 ± 0.00	< 0.00
-0.63	88.00	-0.09 ± 0.00	-0.08 ± 0.00	< 0.00
-0.13	94.00	-0.09 ± 0.00	-0.09 ± 0.00	< 0.00
-0.06	94.00	-0.09 ± 0.00	-0.09 ± 0.00	< 0.00
-0.53	88.00	-0.09 ± 0.00	-0.09 ± 0.00	< 0.00
-0.30	84.00	-0.08 ± 0.00	-0.08 ± 0.00	< 0.00

All numerical observations have been reported up to two decimal digits.

Table S3. Ion current (Mean ± Std. Error, AU) pertaining to significant CV-DF combinations (first ten) for LCD/X-disease symptomatic and asymptomatic samples for cultivar: ‘Cristalina’.

**CV**	**DF**	**Asymptomatic**	**Symptomatic**	***p*** **-value**
0.20	98.00	-0.08 ± 0.00	-0.09 ± 0.00	< 0.00
0.17	98.00	-0.08 ± 0.00	-0.09 ± 0.00	< 0.00
0.22	98.00	-0.08 ± 0.00	-0.09 ± 0.00	< 0.00
0.15	98.00	-0.08 ± 0.00	-0.09 ± 0.00	< 0.00
0.43	96.00	-0.08 ± 0.00	-0.09 ± 0.00	< 0.00
-0.37	98.00	-0.08 ± 0.00	-0.08 ± 0.00	< 0.00
-0.39	98.00	-0.08 ± 0.00	-0.09 ± 0.00	< 0.00
0.29	90.00	-0.06 ± 0.00	-0.08 ± 0.00	< 0.00
0.45	96.00	-0.07 ± 0.00	-0.09 ± 0.00	< 0.00
0.27	90.00	-0.08 ± 0.00	-0.08 ± 0.00	< 0.00

All numerical observations have been reported up to two decimal digits.

Table S4. Ion current (Mean ± Std. Error, AU) pertaining to significant CV-DF combinations (first ten) for LCD/X-disease symptomatic and asymptomatic samples for cultivar: ‘Tieton’.

**CV**	**DF**	**Asymptomatic**	**Symptomatic**	***p*** **-value**
-0.72	84.00	-0.08 ± 0.00	-0.07 ± 0.00	< 0.00
-0.70	84.00	-0.08 ± 0.00	-0.07 ± 0.00	< 0.00
-0.37	98.00	-0.09 ± 0.00	-0.08 ± 0.00	< 0.00
-0.67	84.00	-0.08 ± 0.00	-0.07 ± 0.00	< 0.00

-0.34	98.00	-0.09 ± 0.00	-0.08 ± 0.00	< 0.00
-0.72	94.00	-0.08 ± 0.00	-0.07 ± 0.00	< 0.00
-0.39	98.00	-0.09 ± 0.00	-0.08 ± 0.00	< 0.00
-0.70	94.00	-0.08 ± 0.00	-0.08 ± 0.00	< 0.00
-0.60	88.00	-0.08 ± 0.00	-0.08 ± 0.00	< 0.00
-0.63	94.00	-0.09 ± 0.00	-0.08 ± 0.00	< 0.00

All numerical observations have been reported up to two decimal digits.

Table S5. Significant compensation voltage and dispersion field combinations for LCD/X-disease detection.

**Cultivar**	**Number of significant combinations**	
	$p < 0.05$	$p < 0.01$
Benton	10335	2742
Cristalina	2659	622
Tieton	8874	4613

Table S6. ANOVA for intra- and inter-specific variations in the ion current spectra for all tested cultivars. S: symptomatic; AS: asymptomatic.

**Sample type**	**Source of Variation**	**SS**	**df**	**MS**	**F**	***p*-value**	**F crit**
S	Between Groups	3.55	2	1.77522	11.103	1.51E-05	2.99
	Within Groups	12524.4	78333	0.159887			
	Total	12527.95	78335				
AS	Between Groups	10.45	2	5.22	34.81	7.77E-16	2.99
	Within Groups	11749.25	78333	0.15			
	Total	11759.69	78335				
